# The headache under-response to treatment (HURT) questionnaire, an outcome measure to guide follow-up in primary care: development, psychometric evaluation and assessment of utility

**DOI:** 10.1186/s10194-018-0842-6

**Published:** 2018-02-14

**Authors:** T. J. Steiner, D. C. Buse, M. Al Jumah, M. L. Westergaard, R. H. Jensen, M. L. Reed, L. Prilipko, F. S. Mennini, M. J. A. Láinez, K. Ravishankar, F. Sakai, S.-Y. Yu, M. Fontebasso, A. Al Khathami, E. A. MacGregor, F. Antonaci, C. Tassorelli, R. B. Lipton, on behalf of Lifting The Burden: The Global Campaign against Headache

**Affiliations:** 1 Department of Neuromedicine and Movement Science, NTNU Norwegian University of Science and Technology, NO-7941 Trondheim, Norway; 2 Division of Brain Sciences, Imperial College London, London, UK; 3 Department of Neurology, Albert Einstein College of Medicine, Bronx, NY USA; 4 Montefiore Headache Center, Montefiore Medical Center, Bronx, NY USA; 5 King Abdullah International Medical Research Center, King Saud Bin Abdulaziz University for Health Sciences, National Guard Health Affairs, Riyadh, Saudi Arabia; 6 Danish Headache Centre, Department of Neurology, Rigshospitalet Glostrup University of Copenhagen, Glostrup, Denmark; 7 Vedanta Research, Chapel Hill, NC USA; 8 Department of Mental Health and Substance Abuse, World Health Organization, Geneva, Switzerland; 9 CEIS EEHTA, Faculty of Economics, University of Rome “Tor Vergata”, Rome, Italy; 10 Institute of Leadership and Management in Health, Kingston University, Kingston upon Thames, UK; 11 Department of Neurology, University Clinic Hospital, Catholic University of Valencia, Valencia, Spain; 12 The Headache and Migraine Clinic, Jaslok Hospital and Research Centre, Mumbai, India; 13 Lilavati Hospital and Research Centre, Mumbai, India; 14 Saitama International Headache Center, Tokyo, Japan; 15 Department of Neurology, Chinese PLA General Hospital, Bejing, People’s Republic of China; 16 Headache Expert, Author and Headache Education Facilitator, York, UK; 17 Centre for Neuroscience & Trauma, Blizard Institute of Cell and Molecular Science, London, UK; 18 Headache Science Centre, C Mondino National Neurological Institute, Pavia, Italy; 19 Department of Brain and Behavioural Sciences, University of Pavia, Pavia, Italy

**Keywords:** Headache disorders, Migraine, Tension-type headache, Cluster headache, Medication-overuse headache, Management, Treatment planning, Treatment optimization, Acute treatment, Preventative treatment, Treatment satisfaction, Assessment, Burden, Instruments, Outcome measures, HURT questionnaire, Follow-up, Primary care, Global campaign against headache

## Abstract

**Background:**

Headache disorders are both common and burdensome but, given the many people affected, provision of health care to all is challenging. Structured headache services based in primary care are the most efficient, equitable and cost-effective solution but place responsibility for managing most patients on health-care providers with limited training in headache care. The development of practical management aids for primary care is therefore a purpose of the Global Campaign against Headache. This manuscript presents an outcome measure, the Headache Under-Response to Treatment (HURT) questionnaire, describing its purpose, development, psychometric evaluation and assessment for clinical utility. The objective was a simple-to-use instrument that would both assess outcome and provide guidance to improving outcome, having utility across the range of headache disorders, across clinical settings and across countries and cultures.

**Methods:**

After literature review, an expert consensus group drawn from all six world regions formulated HURT through item development and item reduction using item-response theory. Using the American Migraine Prevalence and Prevention Study’s general-population respondent panel, two mailed surveys assessed the psychometric properties of HURT, comparing it with other instruments as external validators. Reliability was assessed in patients in two culturally-contrasting clinical settings: headache specialist centres in Europe (*n* = 159) and primary-care centres in Saudi Arabia (*n* = 40). Clinical utility was assessed in similar settings (Europe *n* = 201; Saudi Arabia *n* = 342).

**Results:**

The final instrument, an 8-item self-administered questionnaire, addressed headache frequency, disability, medication use and effect, patients’ perceptions of headache “control” and their understanding of their diagnoses. Psychometric evaluation revealed a two-factor model (headache frequency, disability and medication use; and medication efficacy and headache control), with scale properties apparently stable across disorders and correlating well and in the expected directions with external validators. The literature review found few instruments linking assessment to clinical advice or suggested actions: HURT appeared to fill this gap. In European specialist care, it showed utility as an outcome measure across headache disorders. In Saudi Arabian primary care, HURT (translated into Arabic) was reliable and responsive to clinical change.

**Conclusions:**

With demonstrated validity and clinical utility across disorders, cultures and settings, HURT is available for clinical and research purposes.

**Electronic supplementary material:**

The online version of this article (10.1186/s10194-018-0842-6) contains supplementary material, which is available to authorized users.

## Background

Headache disorders are very common, affecting half or more of all adults and many children worldwide [1], and they are highly burdensome [2–8]. The World Health Organization (WHO) recognised them nearly 20 years ago as a global public-health priority [9]. Among them, migraine and tension-type headache (TTH) are often lifelong illnesses. Medication-overuse headache (MOH), usually a complication of migraine or TTH caused by mistreatment of one or the other, is highly frequent, often present daily or almost so. These three headache disorders are the substantial contributors to public ill health, although there are many others [10]. They cause pain, hinder work, damage family and social relationships and impoverish quality of life (QoL).

Headache disorders are largely treatable [11], and they demand treatment not only because it is inhumane to ignore these burdens of ill health. The high levels of disability associated with headache [2–8] lead to very large productivity losses, so that these disorders are very costly in a financial sense [12, 13]. Although the economic consequences of improving headache care are not well studied empirically [14], health services that make the effective remedies more widely available would very probably be *cost saving* in most economies [12, 15]. Treatment of headache is therefore demanded also because it is economically sensible.

Nevertheless, given the very large numbers of people affected, adequate provision of health care to everyone for whom self-care is insufficient – perhaps 50% of those with a headache disorder [16] – is highly challenging.

Structured headache services, with their basis in primary care and supported by educational initiatives, are the most efficient, equitable and cost-effective solution [12]. Such services place responsibility for managing most people with headache on health-care providers who lack special expertise in headache management [16]. This is not in itself problematic: medical management of headache disorders, for the great majority of people affected by them, requires no specialist expertise or investigations but only the diagnostic and management skills that are available to all physicians and many other health-care providers in primary care [12, 16]. On the other hand, non-specialists throughout the world may have received limited training in the application of these basic skills to headache diagnosis and treatment [12].

The development of practical management aids for primary care therefore became an early objective of the Global Campaign against Headache [17–19], a programme of action for the benefit of people with headache conducted by the UK-registered non-governmental organization *Lifting The Burden* (LTB) in official relations with WHO [20]. This manuscript presents one such aid, the Headache Under-Response to Treatment (HURT) questionnaire; it describes its purpose and its development, and summarises the studies undertaking its psychometric evaluation and assessment for clinical utility.

### Objective

We believed that a simple outcome measure was insufficient. Whenever treatment of a patient is started, or changed, follow-up should either ensure that treatment is optimal or recognise that it is not and identify the change(s) in treatment needed. Evaluation of outcome should therefore be coupled with guidance to improve outcome. While this was the instrument’s perceived purpose, it is not so simple a purpose – resources, services and expectations vary greatly between countries and cultures – and no other treatment-assessment instrument met it [21]. No previously described instrument had been shown to function for all important headache disorders or across cultures, and no single instrument covered the range of assessment and decision-making necessary for successful headache management [21]. Few made the key link between assessment and clinical advice, essential because, even in optimal circumstances, outcomes are rarely perfect. Judgement may be needed as to whether the outcome achieved in an individual patient is the best that the patient can reasonably expect. For the non-specialist in particular, one question sometimes arising is: “What further effort, in hope of a better outcome, is justified?” A second question, which may follow, is “What is it that needs changing?”

The objective, therefore, was an instrument that would both assess outcome and provide answers to these two questions, offering guidance on appropriate actions towards treatment optimisation. More specifically, we aimed to create a short, simple-to-use management aid, versatile yet reliable and sensitive, which met the purpose described and three utility requirements:

1. in both primary and specialist care;
2. across the range of headache disorders of public-health importance;
3. across countries and cultures, despite wide variation in resources, services and expectations.

This objective would be reached through a development process invoking expert consensus followed by psychometric and clinical evaluations involving professional and lay representatives of all world regions.

## Methods

Development was a multi-stage process including item development, item reduction using item-response theory (IRT), psychometric testing and external validation, and assessment of clinical utility.

For item development, LTB brought together an international expert consensus group (Table 1), with the technical support of WHO. The members, from all six world regions, included headache specialist health-care professionals, primary-care physicians, lay patient advocates and experts in health economics, psychometrics and qualitative research. The group met first in April 2006 at WHO headquarters in Geneva and agreed the domains that the instrument should address. After a literature review of existing instruments with related purposes, a large item pool within these domains was developed. In further discussions within the group, and through consensus, initial item selection was followed by a process of item reduction, refining the selection and limiting the number of items to those considered most apt (relevant and informative). These candidate items were formulated into simple questions, with response options based on the group’s expert knowledge and clinical experience, and included in the emerging instrument. A scoring system was constructed around them, with guidance on interpretation. Accompanying clinical advice, according to responses, was developed through reference to authoritative treatment guidelines from multiple countries coupled again with the group’s expert input. A design process then built a first version (“HURT-v1”) of the end-product as a 2-page document.

**Table 1:** Development group

Convener: TJ Steiner (UK) *Meeting in Geneva* RB Lipton (USA) (*chairman*), M Al Jumah (Saudi Arabia), B Eggleston (Australia) (lay), M Fontebasso (UK), L Gardella (Argentina), RH Jensen (Denmark), H Kettinen (Finland) (lay), MJA Láinez (Spain), FS Mennini (Italy), M Peters (UK), L Prilipko (WHO), K Ravishankar (India), F Sakai (Japan), N Sharma (India) (lay), TJ Steiner (UK), AD Tehindrazanarivelo (Madagascar), D Valade (France), S-Y Yu (China) Contributing remotely W Stewart (USA), M Von Korff (USA) *Meeting in New York* RB Lipton (USA) (*chairman*), M Al Jumah (Saudi Arabia), S Broner (USA), DC Buse (USA), ML Reed, (USA), D Serrano (USA), M Fontebasso (UK), FS Mennini (Italy), TJ Steiner (UK) *Contributing remotely* RH Jensen (Denmark)	

The Geneva group’s final task was to name the instrument.

Psychometric evaluation (see below) was carried out on HURT-v1. Informed by the results, a smaller group met in New York City in October 2007 (Table 1), making changes as necessary, again through consensus, and generating the final version (“HURT-final”). Clinical evaluations were performed on HURT-final.

### Evaluation

Two groups were involved in evaluation of HURT, one in psychometric testing and the other in clinical assessments (Table 2).

**Table 2:** Evaluation groups

*Psychometric evaluation* M Al Jumah (Saudi Arabia), A Al Khathami (Saudi Arabia), A Al Owayed (Saudi Arabia), DC Buse (USA), A Jawhary (Saudi Arabia), RH Jensen (Denmark), S Kojan (Saudi Arabia), RB Lipton (USA), EA MacGregor (UK), ML Reed (USA), D Serrano (USA), CM Sollars (USA), TJ Steiner (UK), H Tamim (Saudi Arabia), ML Westergaard (Philippines/Denmark) *Clinical evaluation* M Al Jumah (Saudi Arabia), A Al Khathami (Saudi Arabia), A Al Owayed (Saudi Arabia), F Antonaci (Italy), DC Buse (USA), A Jawhary (Saudi Arabia), RH Jensen (Denmark), S Kojan (Saudi Arabia), RB Lipton (USA), EA MacGregor (UK), TJ Steiner (UK), H Tamim (Saudi Arabia), C Tassorelli (Italy), ML Westergaard (Philippines/Denmark)	

### Psychometric testing and validation

The American Migraine Prevalence and Prevention (AMPP) Study [22] recruited from a panel constructed and maintained by the survey company NFO, Inc. (Ames, IA, USA) [23] to match the US population, according to the national census, by age, gender, socioeconomic status and region of the country. The AMPP screening survey identified participants describing themselves as affected by “severe headache”, a term intended preferentially to capture those with migraine and/or with unmet needs for headache treatment. We included HURT-v1 in two surveys mailed to samples of these AMPP Study participants.

### Ethics approval

The AMPP Study, with inclusion of HURT, was approved by the Albert Einstein College of Medicine Institutional Review Board. The mailings included explanations of the study, and voluntary participation in the surveys implied consent.

### Sociodemographic variables

Recorded in these surveys were age, gender, weight and height, all self-reported. Body-mass index (BMI) was calculated from height and weight using the WHO standard formula. Data were also collected on average annual household income, race and geographic region.

### US mailing #1: Psychometric testing

HURT-v1, including all candidate items and response options, was posted to a first sample (*N* = 2500) in summer, 2006. Enclosed in the mailing were question sets on the sociodemographic variables and on headache frequency, severity, associated symptoms and treatment.

The headache-related data were used to diagnose participants with migraine, using the American Migraine Study (AMS)/AMPP Study diagnostic module (demonstrated to have 100% sensitivity and 82% specificity for this diagnosis) [24]. Case-definition criteria for migraine accorded with ICHD-II [25], except that a lifetime history of ≥5 attacks could not be ascertained from the cross-sectional data. No significant changes have occurred between ICHD-II [25] and ICHD-3-beta [10] with regard to these criteria. Only participants with headache on ≤14 days/month were included as migraine cases (*ie*, episodic migraine) in this analysis.

### Item analysis

Responses from these participants to HURT items 1–6 were included in a graded item-response model applied to assess scale properties using SAS NLMIXED and to identify an underlying latent variable. However, the HURT metrics for items 1–6 varied: four were count items (with two based on 1 month’s recall and two based on 3 months’ recall) and two were polytomous response items. To simplify item analysis, we harmonized item responses, placing all on an identical metric. Count items were converted to five-category polytomous items, with severity values in the range 0–4 as follows:

1. 0 (very mild): 0 days in 1 month or 0 days in 3 months;
2. 1 (mild): 1–2 days in 1 month or 1–5 days in 3 months;
3. 2 (moderate): 3–5 days in 1 month or 6–10 days in 3 months;
4. 3 (severe): 6–15 days in 1 month or 11–20 days in 3 months;
5. 4 (very severe): ≥16 days in 1 month or ≥21 days in 3 months.

The original two polytomous response items were harmonized so that higher-ordered response options were also indicative of greater severity. Consequently, the medication efficacy item was reverse-coded (0–4) to reflect increasing *inefficacy*, and the headache-control item was reverse-coded (0–4) to reflect increasing *lack of* control. Total and factor-specific scores could then be computed as simple sums of the scale items.

An item-response model was fitted to these item scores using a multinomial response-distribution. Item parameters were estimated for the five response categories offered for each item. A confirmatory factor analysis model was also employed where the original count items loaded on the first factor and the original polytomous items loaded on the second. The correlation between the two factors was freely estimated.

### Performance across headache types

In the second mailing sample (described below), we attempted to contrast HURT factor scores across headache types to assess relative performance.

### US mailing #2: External validity

HURT-v1 was posted, in autumn 2006, to a different sample (*N* = 2250), along with similar sociodemographic and headache question sets. Included in this mailing were multiple external validators: other instruments used in headache management and themselves well-validated as measures of headache-attributed impact, treatment optimization, health-related QoL (HRQOL) or comorbid depression. These are described in Table 3. For purposes of correlation with these external validators, we used the total HURT scores derived during item analysis (see above). Comparisons employed Spearman correlations.

**Table 3:** Instruments used as external validators

Instrument	Structure/purpose	Scoring
Migraine Disability Assessment (MIDAS) scale [29]	5 items counting days of lost productivity (complete or by > 50%) from paid or household work and missed family, social or leisure activities during the preceding 3 months	Sum of responses (the MIDAS score) quantifies impact in days lost per 3 months
Headache Impact Test (HIT-6) [30]	6 items assessing lost time at work, school or social activities, pain severity, fatigue, frustration and difficulty in concentration, each with 5 frequency response options (never, rarely, sometimes, very often, always)	Response options scored 6, 8, 10, 11 and 13 respectively, with higher summed scores (range 36–78) indicating greater impact
Migraine Specific Quality of Life (MSQ) questionnaire, version 2.1 [32]	14 items assessing QoL in the preceding 4 weeks in 3 migraine-specific dimensions (role function-restrictive; role function-preventive; emotional function), each item having 6 Likert-type response options.	On a scale 0–100 (higher being better QoL) through transformation involving 3 steps
PRIME-MD Patient Health Questionnaire – depression module (PHQ-9) [31]	Depressive symptoms over the preceding 2 weeks assessed in the 9 domains of DSM-IV, each with 4 frequency response options (not at all, on several days, on more than half of days, nearly every day)	Response options scored 0–3, with higher summed scores (range 0–27) indicating more severe depression
Migraine Prevention Questionnaire (MPQ) [33]	5 items (regarding headache frequency, acute medication use and headache-related impairment, worry and anxiety) identify need for, and guide, preventative pharmacological treatment of migraine based on consensus guidelines	Responses summed into a total score, which falls into one of 3 categories: preventative treatment not indicated, should be considered or should be offered

### Reliability

Two studies were undertaken to assess reliability, in very different cultural and clinical settings. Both have been published previously [26, 27], and are therefore only summarised here.

HURT-final was completed twice by 159 consecutive patients seeking non-urgent care in headache specialist centres in Denmark and United Kingdom (UK) [26]. The instrument was first mailed to patients as they joined the clinic waiting lists, before their initial visits, and then given to them at these initial visits, which were expected usually to take place about 1 month later.

For testing in Arabic-speaking patients in Saudi Arabia, HURT was translated according to LTB’s translation protocol for hybrid documents [28]. Test-retest reliability was assessed in 40 consecutive patients of four primary-care centres, who completed HURT at two visits 4–6 weeks apart while receiving usual care [27].

### Ethics approval

In Denmark and UK, evaluation of HURT as an outcome measure was considered a service-improvement project, falling outside the scope of research ethics review [26]. Ethics approval was requested in Saudi Arabia, and granted by the Institutional Review Board of National Guard Health Affairs [27]. In both studies, information was provided and all participants gave consent.

### Clinical testing

The first assessment of clinical utility, to demonstrate that HURT was responsive to change induced by effective management, was conducted in headache specialist centres in three European countries [26]. In Denmark and UK, HURT was administered on a third occasion to the same 159 consecutive patients when, in each case, the treating specialist judged that the best possible outcome had been achieved. In Italy, HURT was answered by 42 patients at initial and final visits [26].

### Clinical testing

The second assessment evaluated the Arabic version of HURT in primary care in Saudi Arabia. The general practitioners (GPs) in four centres were trained in headache management, and the centres then randomized in pairs to control (standard care) or intervention (care guided by implementation of HURT). Responsiveness of HURT to clinical change was assessed by comparing base-line responses to HURT questions 1–6 with those at follow up. Clinical utility was assessed by comparing outcomes between control and intervention pairs after 3 months, using locally-developed 5-point verbal-rating scales: patient-satisfaction scale (PSS) and doctor-satisfaction scale (DSS) [27].

### Ethics approval

In Denmark and UK, these studies again fell outside the scope of research ethics review [26]. Ethics approvals were obtained in Italy from the ethics review committee of C Mondino Foundation, University of Pavia [26], and in Saudi Arabia from the Institutional Review Board of the King Abdullah International Medical Research Center (KAIMRC) [27]. In both studies, information was provided and all participants gave consent.

## Results

The Geneva meeting identified the following as essential domains: headache frequency, disability, medication use and effect, patients’ perceptions of headache “control” and their understanding of their diagnoses. The group developed an 8-item self-administered questionnaire addressing these. Responses were either numerated in days over a one- or three-month recall-period or selected from Likert options. In either case, responses were graded into an area of “no concern” or one of three flagged areas indicating increasingly important treatment deficiencies; clinical advice was provided for each of the latter.

The design process constructed the questionnaire, with responses, interpretation and advice, into the form shown (Additional file 1).

After the psychometric evaluations, and informed by them, the New York meeting made only minimal changes to generate HURT-final.

### Psychometric evaluation

#### Psychometric testing

In the assessment of scale properties, valid returns came from 1691 (68%) respondents to the first US mailing. A large majority (1362; 81%) met ICHD-II criteria for migraine [25]. IRT revealed a two-factor model: 1) headache frequency, disability and medication use; and 2) medication efficacy and headache control items. Total and sub-factor scores correlated strongly (Spearman coefficient r = 0.49–0.86).

#### Performance across headache types

In the second US mailing sample, which, like the first, were self-selecting for “severe” headache, sub-sample sizes for headache types other than episodic migraine were very low (≤100): 73 cases met criteria for episodic TTH (ETTH); 100 had headache on ≥15 days/month, with ten of these diagnosed as chronic migraine. Several simplifying modifications were attempted in order to contrast other headache types with episodic migraine, but all models failed in the face of these small samples and insufficient information. Furthermore, respondents with headache on ≥15 days/month inevitably reported a constant (maximal) value on HURT item 1 (headache days/month). As a last resort, fitting the item-response model separately to the episodic migraine sub-sample and the headache on ≥15 days/month sub-sample (the only other for which the model contrast was estimable), we compared the estimated factor scores for these two groups in an ANOVA (Table 4). No significant differences were observed in factor scores for the two factors, suggesting no meaningful difference in the performance of HURT between these headache types.

**Table 4:** Estimated factor scores for episodic migraine sub-sample versus headache on ≥15 days/month sub-sample

Source	DF	Type III sum of squares	Mean square	F	Pr > F
Migraine	1	0.5951	0.5951	0.78	0.3763
Effect	1	0.1050	0.1050	0.14	0.7101
Migraine*Effect	1	0.1579	0.1579	0.21	0.6486

#### External validity

Valid returns to the second mailing were received from 1734 (69%) respondents, of whom 1391 (80%) met ICHD-II criteria for migraine [25]. Summed HURT scores correlated in the expected directions with the other measures. Correlations were positive with both the MIDAS questionnaire (r = 0.69) and HIT-6 (r = 0.49), instruments that are strongly influenced by disability [29, 30], and with PHQ-9 (r = 0.34), a measure of depression [31]. Correlations were negative with MSQ version 2.1, a migraine-specific QoL instrument [32], so that higher HURT scores were associated with poorer QoL (restrictive subscale: r = − 0.53; preventative subscale: r = − 0.58; emotional subscale: r = − 0.51). HURT scores correlated most strongly, and positively, with MPQ (r = 0.86), an instrument indicating need for preventative pharmacological treatment of migraine [33]. These correlations suggested that, while the instruments shared some variance, they nonetheless captured unique constructs.

#### Reliability

Test-retest reliability in specialist care in Denmark and UK was fair to low (κ = 0.38–0.62; r = 0.49–0.76) [26]. It should be noted that, while the interval between first and second applications of HURT was intended in the study design to be about 1 month, subsequent review of the Danish records revealed a range between 1 day and 9 months (median 1.7 months). The longer-than-planned intervals were caused in part by the centre’s extending waiting time but also in part by patients who changed appointment dates – sometimes introducing long delays. Internal consistency reliability on the other hand was very good (Cronbach’s α = 0.85) [26]. In Saudi Arabian primary care, intra-class correlation coefficients were 0.66–0.78 (all *p* ≤ 0.001) for questions 1–4 and 0.90–0.93 for questions 5–7 (all *p* < 0.0001) [27]. For the dichotomous response to question 8, κ = 1 (p < 0.0001). Internal consistency reliability was good (Cronbach’s α = 0.74) [27].

### Clinical evaluation

In specialist-care utility evaluation in 201 patients in Europe, with HURT administered at the start and end of treatment, good outcomes were judged to have been achieved in 155 (77%) [26]. HURT reflected these in significant differences between recorded responses at base line and post-intervention (*p* < 0.01), indicating responsiveness of the instrument to change. Analyses of paired responses (before and after treatment) found seven of the eight HURT items indicating improvements (*p* < 0.001). No evident improvement in patients’ concerns about side-effects of medication was indicated by item 7 (*p* = 0.18) [26].

In 342 patients in Saudi Arabian primary care, HURT signalled clinical improvement over 3 months through statistically significant reductions (*p* < 0.0001) in responses to each of questions 1–6 [27]. HURT also distinguished clearly between patients with good or poor outcomes, with questions 1–4 each (p < 0.0001) indicating improvement in those reporting satisfaction (positive PSS scores) and worsening (or no improvement) in those reporting dissatisfaction (negative PSS scores) [27]. There was a trend (*p* = 0.06) towards greater patient-satisfaction among those treated with guidance from HURT (*n* = 207) than among controls (*n* = 135). There was a slight opposite trend in doctor-satisfaction [27].

## Discussion

HURT is a consensus-developed 8-item self-administered questionnaire addressing headache frequency, disability, medication use and effect, patients’ perceptions of headache “control” and their understanding of their diagnoses. Its scope is unique among outcome measures.

In the evaluations, HURT proved to be a psychometrically sound instrument for monitoring headache treatment, with, importantly, scale properties apparently stable across headache disorders. While test-retest reliability in specialist care was assessed as fair to low, this was, very probably, because headache in this setting, and prior to start of management, was unstable over time. Although this was not intended, intervals of up to 9 months between the two applications of HURT allowed the possibility of real change in many patients. In the very different setting of Saudi Arabian primary care, reliability was good for questions 5 to 7, to which responses expressed beliefs or perceptions at the time of testing. It was less good for questions 1 to 4, which required recall of past events over 1 to 3 months and which, again, were subject to real changes in the frequencies of events potentially occurring over the test-retest period. It should be noted also that, unlike in Europe, patients in Saudi Arabia did receive an intervention (albeit only standard care) between the two administrations of HURT. The two settings were, therefore, not comparable for testing reliability (it was not intended that they should be). In USA, summed scores correlated well and in the expected directions with external validation measures.

The literature search yielded a multiplicity of psychometrically robust instruments available to support the several key steps in comprehensive care of headache disorders [21]. These instruments can be used to aid the diagnosis of primary headache disorders; the exclusion of secondary headaches; the assessment of headache symptoms and their severity, headache-attributed disability, burden and impact (both ictal and interictal); the recognition and assessment of comorbidities, and of psychological factors shown to predict adherence to treatment and outcomes; outcome measurement including patients’ satisfaction; and treatment planning, monitoring and optimization for both acute and preventative pharmacological therapies. Many have particular strengths. MIDAS in particular, although it measures headache-attributed lost time rather than disability [34], is scored in meaningful units, is easy to use, has demonstrated reliability and validity, predicts treatment needs, has been extensively used in clinical practice in many countries and is sensitive to change, at least in severely-affected patients. However, no single instrument other than HURT combines so many of these aspects of care, and, especially, few instruments link assessment to clinical advice or suggested actions [21]. Thus, they do not serve non-headache experts well, especially health-care providers in primary care.

The HURT questionnaire was created to fill this gap and it appears to have the capability of doing so.

In Europe, HURT showed utility in specialist care as an outcome measure across headache disorders. In this setting, where many patients are partially or wholly refractory even to best care, HURT can be used to define and explain treatment goals. In addition, it can promote self-efficacy and knowledge about headache. In Saudi Arabian primary care, where no other Arabic instruments were available as standards for comparison, HURT was reliable and responsive to clinical change and therefore it has clinical utility in Arabic-speaking patients. One explanation of the apparent discord between doctors’ and patients’ satisfaction in Saudi Arabia is that the instrument worked as it should – improving care (perceived by patients) while revealing deficiencies in care and need for improvement to the physicians.

## Conclusions

The HURT questionnaire has demonstrated validity and clinical utility, and is currently available for clinical use and research purposes in English and Arabic. Some other translations are known to exist, and future work may include use and evaluation in other countries and cultures.

## Additional file

Additional file 1: HURT questionnaire. (PDF 105 kb)

## Abbreviations

AMPP: American Migraine Prevalence and Prevention Study
ANOVA: analysis of variance
DSS: doctor-satisfaction scale
GP: general practitioner
HIT-6: Headache Impact Test
HURT: Headache Under-Response to Treatment
ICHD: International Classification of Headache Disorders
IRT: item-response theory
LTB: *Lifting The Burden*
MIDAS: Migraine Disability Assessment scale
MOH: medication-overuse headache
MPQ: Migraine Prevention Questionnaire
MSQ: Migraine Specific Quality of Life questionnaire
PHQ: PRIME-MD Patient Health Questionnaire
PSS: patient-satisfaction scale
QoL: quality of life
TTH: tension-type headache
UK: United Kindom
USA: United States of America
WHO: World Health Organization
